# Do oral bacteria alter the regenerative potential of stem cells? A concise review

**DOI:** 10.1111/jcmm.12613

**Published:** 2015-06-08

**Authors:** Kyriaki Chatzivasileiou, Katja Kriebel, Gustav Steinhoff, Bernd Kreikemeyer, Hermann Lang

**Affiliations:** a Department of Operative Dentistry and Periodontology, University of RostockRostock, Germany; b Institute of Medical Microbiology, Virology and Hygiene, University of RostockRostock, Germany; c Department of Cardiac Surgery, University of RostockRostock, Germany

**Keywords:** stem cells, bacteria, regeneration, inflammation, periodontitis

## Abstract

Mesenchymal stem cells (MSCs) are widely recognized as critical players in tissue regeneration. New insights into stem cell biology provide evidence that MSCs may also contribute to host defence and inflammation. In case of tissue injury or inflammatory diseases, *e.g*. periodontitis, stem cells are mobilized towards the site of damage, thus coming in close proximity to bacteria and bacterial components. Specifically, in the oral cavity, complex ecosystems of commensal bacteria live in a mutually beneficial state with the host. However, the formation of polymicrobial biofilm communities with pathogenic properties may trigger an inadequate host inflammatory-immune response, leading to the disruption of tissue homoeostasis and development of disease. Because of their unique characteristics, MSCs are suggested as crucial regulators of tissue regeneration even under such harsh environmental conditions. The heterogeneous effects of bacteria on MSCs across studies imply the complexity underlying the interactions between stem cells and bacteria. Hence, a better understanding of stem cell behaviour at sites of inflammation appears to be a key strategy in developing new approaches for *in situ* tissue regeneration. Here, we review the literature on the effects of oral bacteria on cell proliferation, differentiation capacity and immunomodulation of dental-derived MSCs.

## Introduction

Mesenchymal stem cells (MSCs), also referred to as multipotent mesenchymal stromal cells, are present in all the organs throughout the body and play a key role in tissue regeneration. Aside from their ability to orchestrate regeneration processes, MSCs are newly proposed as critical players in host defence and inflammation 1.

Under physiological conditions, the oral cavity, gastrointestinal tract and skin home complex ecosystems of commensal bacteria that live in a mutually beneficial state with the host. In case of tissue injury or inflammatory diseases, stem cells are mobilized towards the site of damage, thus coming to close vicinity of bacteria and bacterial components. Bacterial infection of stem cells could even lead to long-term functional consequences for the host 2. Recent reports suggest that MSCs may be able to actively participate in the control of infectious challenges by direct targeting of bacteria and through indirect effects on the host primary and adaptive immune response 3.

## Oral microflora

The oral cavity, like the skin, the respiratory tract and the gut, is habitat to a plethora of microbiota living in symbiosis with the host 4. The oral microflora is known to contain over 700 species of aerobic and anaerobic bacteria 5. These organisms can be isolated from tooth surfaces, periodontal pockets and other oral sites such as the tongue and oral mucous membranes 6. Oral microbiota grow as complex, mixed, interdependent colonies organized in biofilms 7. Reports in the literature suggest that oral bacterial biofilms may contain more than 10^5^ microorganisms 8, while the concentrations and compositions of pathogenic bacteria in the subgingival biofilm vary greatly depending on the local microenvironmental conditions 9,10. The bacterial genera which are mostly represented in the oral cavity include the following: *Gemella*, *Granulicatella*, *Streptococcus*, *Veillonella*, *Neisseria*, *Haemophilus*, *Rothia*, *Actinomyces*, *Prevotella*, *Capnocytophaga*, *Porphyromona*, *Fusobacterium*, *Corynebacterium*, *Cardiobacterium*, *Campylobacter*, *Corynebacterium*, *Atopobium* and *Bergeyella* 11–13. It should be noticed that almost 60% of the species detected by new molecular methods are not presently cultivable and remain uncharacterized 11.

The natural oral microflora is vital for the normal development and physiological integrity of the oral cavity. It also contributes to host defence by excluding exogenous microorganisms 14. It is widely recognized that the maintenance of an ecologically balanced biodiversity of the microflora within the oral cavity is crucial not only to the oral health but also to the general health of the host 15. Microbes have commensal relationships with their co-habitants, while being symbiotic with their host 16. However, ecological shifts may lead to pathological conditions, which alter the relationships between microbes and the host 17. In disease, pathogenic bacteria grow with disregard to their co-habitant bacteria and express their virulence properties, so that the host becomes infected or susceptible to infection 16.

## Periodontitis

Periodontitis is a bacterially induced inflammatory disease of the supporting tissues of the teeth. It represents one of the major dental diseases that affect human populations worldwide at high prevalence rates and has a huge economic impact on national health care systems 18. In fact, periodontitis is characterized by progressive periodontal tissue destruction that may finally lead to the loosening and subsequent loss of teeth 19. The predominant pathogens involved in periodontitis are *Aggregatibacter actinomycetemcomitans*, *Porphyromonas gingivalis*, *Prevotella intermedia*, *Fusobacterium nucleatum*, *Tannerella forsythia*, and *Eikenella corrodens*, and *Treponema denticola* 15.In addition, several forms of uncultivable spirochetes are supposed to play a major role in the pathogenesis of this disease 20.

Periodontal pathogens induce tissue destruction by activating the host defence. The infection of periodontal tissues is accompanied by the release of bacterial leucotoxins, collagenases, fibrinolysins and other proteases that break down host tissues and may result in gingival inflammation 21. Specifically, microbial components, like lipopolysaccharide (LPS), have the capacity to activate macrophages and lymphocytes to synthesize and secrete a wide array of molecules including cytokines, prostaglandins, hydrolytic enzymes and tumour necrosis factor alpha, which in turn stimulate the effectors of periodontal tissue breakdown 22. Cell activation occurs mainly through two members of the Toll-like receptor (TLR) family, TLR2 and TLR4 that are documented as predominant signalling receptors for most bacterial components 23,24.

Once a periodontal pocket forms and becomes colonized by bacteria, the pathologic situation becomes irreversible 18. The conventional periodontal treatment involves the mechanical removal of the pathogenic dental biofilm. Successful clinical outcomes such as probing depth reduction and gain of clinical attachment after treatment are well documented in a plethora of studies 25–27. However, histological analyses of healed periodontal tissues reveal in most of the cases the presence of an epithelial lining along the treated root surfaces of the teeth, instead of true periodontal regeneration 28.

## Dental stem cells

Stem cells are defined by their capacity to self-renew and differentiate into multiple cell lineages. One of the most studied adult stem cell types are MSCs 29. Friedenstein *et al*. first described bone marrow stem cells (BMSCs) as a heterogeneous population of multipotent cells derived from bone marrow aspirates with the ability to adhere to plastic surfaces and form colonies of fibroblast-like cells within the first days of cultivation 30,31. Although MSCs were originally isolated from the bone marrow, similar populations of mesenchymal precursors were isolated from other tissues, including adipose tissue 32, amniotic fluid 33, foetal liver 34 and umbilical cord blood (UCB) 35.

During the last decades, rapid progress in dental research has shed light on the molecular and cellular biology of periodontal tissue development. Recently, multipotent cells have been successfully isolated from several dental tissues including dental pulp 36, dental follicle 37, exfoliated deciduous teeth 38 and the root apical papilla 39. Several *in vitro* and *in vivo* studies on dental stem cells (DSCs) provide evidence of their multipotent character and their key role in periodontal regeneration 40. It has been demonstrated that DSCs have a fibroblast-like morphology and are plastic-adherent. Similar to other stem cell populations, DSCs express several surface markers such as CD10, CD13, CD29, CD44, CD53, CD59, CD73, CD90 and CD105, and do not express CD34, CD45 or HLA-DR 41,42. Demonstration of self-renewal ability and multilineage differentiation capacity are additional indications of their stem cell phenotype. Indeed, it has been proven that DSCs are able to form single cell-derived colonies and differentiate into several lineages, when induced by special media *in vitro* 43.

## Clinical relevance

The identification of DSCs has stimulated interest in the potential use of cell-based therapies as prospective alternatives to existing therapeutic approaches for the repair and regeneration of the periodontium 44. One of the critical requirements for the success of such therapeutic interventions would be the repopulation of the periodontal wound by *ex vivo* expanded progenitor populations or the mobilization of endogenous progenitor cells capable of promoting regeneration 45. Specifically, DSCs grafts may support the restoration of the complex ultrastructure of the periodontal ligament and the dynamic functional relationships of its components. Numerous animal studies have already proved the regenerative potency of these cell populations *in vivo* 46.

However, one of the growing concerns in dental research is the exposure of DSCs to the inflamed microenvironment of periodontal pockets 47. This may affect many cell properties such as self-renewal, differentiation potential, production of cytokines and extracellular matrix compounds secretion. Sorrell and Caplan demonstrated that multipotent cell grafts might trigger regenerative processes not only through direct commitment, but also by infiltrating inflammatory or antigen-presenting cells 48. Such a regenerative microenvironment may impel self-regulated regenerative cascades and limit the area of damage in the inflamed adult tissues 49. Hence, a better understanding of cell behaviour at sites of bacterial infection appears to be a key strategy for the development of new approaches for periodontal regeneration.

## *In vitro* experimental models

The microenvironment of a periodontal pocket is characterized by the constant presence of bacterial biofilms. This condition results in a continuous cross-talk of periodontal tissue cells with a wide variety of oral microorganisms. Further, in periodontitis, several types of host immune cells (*e.g*. neutrophils and macrophages) migrate to the site of inflammation 50. The better understanding of the complex cell–bacteria interactions is essential for the development of successful periodontal therapies. While several *in vivo* models have been already used, the design of an *in vitro* model that could sufficiently mimic the *in vivo* situation of inflamed periodontal tissues remains to be developed 51.

Till now most of the *in vitro* experimental settings are based on the analysis of the LPS effects on cells. Lipopolysaccharide is a major membrane component of Gram-negative bacteria and can be derived from several bacterial species, *e.g. Escherichia coli* or *P. gingivalis* 47,52,53. The easy isolation method and the fact that LPS is responsible for many of the inflammatory responses and pathogenic effects of Gram-negative bacteria are the main arguments for the use of LPS in numerous *in vitro* experiments. Experimental settings using heat-inactivated or sonicated bacteria have also been proposed as models that may correspond to the *in vivo* condition of bacterial infection 54,55. Further methods used for the analysis of cell–bacteria interactions are based on the fact that periopathogenic bacterial pathogens produce a broad array of potential virulence factors apart from LPS that are released into the gingival crevicular fluid 56. Thus, the culture of cells with bacterial pre-conditioned medium or the co-cultivation of cells and bacteria in transwell systems have been used to evaluate the secretion of soluble factors and the activation of cellular downstream cascades by bacteria 57,58. Although many biological effects can be elicited by non-viable bacteria, it is known that some cell responses require the presence of live bacteria 59.

Experimental models utilizing microorganisms in a planktonic state were used to imitate the periodontal infection 60. Nevertheless, such systems may not adequately portray the bacterial challenge conferred by a polymicrobial, biofilm-induced disease, such as periodontitis 61. Thus, *in vitro* multispecies dental biofilm settings have been proposed as laboratory models that better mimic the environment of chronic periodontitis 62–64. Finally, cell invasion is a common strategy of pathogens that facilitates their escape from host immune system, access to nutrients, persistence and spread into tissues 65. Recent studies using viable bacteria have been demonstrated as models for the analysis of host cell invasion processes such as bacterial adherence and internalization by cells 66,67. However, the subgingival bacteria that are closely correlated with periodontitis are mainly anaerobes. The co-culture of these bacteria with oxygen-requiring cells in conventional systems is not possible 68. Therefore, one weak point of the experimental studies on periodontal infection is the fact that most *in vitro* settings are conducted under aerated conditions. Given the fact that aerotolerance of strictly anaerobic pathogens like *P. gingivalis* is very low, the interpretation of such experimental results may not directly reflect the *in vivo* situation 69. Until now only few models have been proposed utilizing direct contact between live obligate anaerobic bacteria and human cell lines under oxygen-free conditions 70,71.

## Influence of oral bacteria on stem cells

### Effects on cell viability and proliferation of stem cells

Cell proliferation is fundamental in tissue homoeostasis and can be controlled by either physiological or pathological conditions. Previous studies have demonstrated that LPS derived from periopathogenic bacteria may induce controversial effects on the proliferation of periodontal ligament fibroblasts 72–74. Currently, the possible effect of bacteria on the proliferative rates of multipotent cells is in the focus of interest of several research groups. Kato *et al*. demonstrated that *P. gingivalis* LPS promoted cell proliferation in periodontal ligament stem cells (PDLSCs) 53. Stimulation of TLR2 also led to enhanced proliferation of adult BMSCs 75. Further, Jiang *et al*. and Buchon *et al*. proposed that intestinal stem cells are able to maintain tissue homoeostasis by increasing their proliferation rates to repair tissue damage at sites of infection through the JAK-STAT signalling pathway 76,77.

On the contrary, according to an *in vitro* study on canine adipose-derived MSCs (ADSCs), gastrointestinal microbes did not induce cell death nor diminished cell proliferation. Previous studies on dental follicle progenitors also demonstrated that cell viability of both dental follicle progenitor cells (DFPCs) and BMSCs was not affected by *P. gingivalis* LPS treatment 47,78. In addition, TLR ligands such as LPS and flagellin do not alter proliferation rates of a newly identified population of pluripotent UCB cells, which are termed as unrestricted somatic stem cells 79. Nevertheless, LPS and extracts from *Streptococcus mutans* treatment were able to inhibit the proliferation of dental pulp stem cells (DPSCs) *in vitro* 80.

These heterogeneous effects of bacteria on the induction or inhibition of cell proliferation across studies could imply the complexity of the underlying mechanisms that rule the interactions between host cells and bacteria. Specifically, cell response to bacterial stimuli seems to be associated with the cell type, bacterial strain and specific bacterial components used in each experimental setting 81.

### Effects on differentiation capacity of stem cells

The ability of stem cells to differentiate into multiple lineages is well documented. Especially the differentiation capacity of DSCs across the osteogenic, chondrogenic, adipogenic and neurogenic lineages has been demonstrated from several research groups in the last years 82,83. Nevertheless, the impact of bacteria on the differentiation capacity of stem cells remains to be explored. In a recent study, Ronay *et al*. demonstrated that infected periodontal granulation tissues harbour cells expressing embryonic stem cell markers, and exhibit osteogenic capacities 84. These results are in accordance with other studies demonstrating elevated alkaline phosphatase (ALP) activity, an early marker for osteogenic differentiation, and calcium deposition after *E. coli* LPS treatment of BMSCs 52.

Nevertheless, an increased ALP activity after LPS treatment may not always lead to formation of mineralized nodules *in vitro*. It is suggested that LPS may partly block the progression of molecular processes involved in osteogenic differentiation 47. Interestingly, Abe *et al*. demonstrated that low concentrations of *P. gingivalis* extracts improve the osteogenic differentiation of human dental pulp-derived cells while high concentrations may inhibit ALP activity and bone sialoprotein gene expression 85. High concentration of sodium butyrate, a major metabolic by-product of anaerobic Gram-negative periodontopathogenic bacteria, could inhibit the osteoblastic differentiation and mineralized nodule formation in an osteoblastic cell line *in vitro* 86. In accordance with these results *P. gingivalis* LPS was shown to suppress the osteoblastic differentiation in both PDLSCs and DFPCs 47,53. Nomiyama *et al*. suggested that Gram-negative bacterial infection might down-regulate the odontoblastic properties of rat pulp progenitor cells after stimulation with *A. actinomycetemcomitans* LPS 87. Treatment with LPS from *P. gingivalis* was shown to impair both ALP activity and the formation of mineral deposits in DPSCs 88. In this context, TLR ligands have been proposed as possible regulators of stem cell differentiation state *in vitro* 78.

Further, *P. gingivalis* fimbriae are proposed as potent inducers of a monocyte/macrophage tumour cell line differentiation, *via* cyclic nucleotide-independent protein kinase C 89. However, *P. gingivalis* fimbriae were proved unable to alter the osteoblastic differentiation and mineralization in long-term mouse calvarial osteoblast cultures 90.

### Effects on the immunomodulatory properties of stem cells

In the last years the immunomodulatory functions of the stem cells have been in the focus of research 91. Accumulating evidence indicated that MSCs may affect neighbouring innate and adaptive immune cells by two main ways: the direct cell–cell contact and the release of a variety of soluble factors 92–97. Gingiva-derived MSCs (GMSCs) were shown to have immunomodulatory functions. Specifically, GMSCs were able to suppress peripheral blood lymphocyte proliferation and induce expression of a wide panel of immunosuppressive factors including interleukin (IL)-10, indoleamine 2,3-dioxygenase, inducible nitric oxide synthase and cyclooxygenase 2 in response to the inflammatory cytokine, interferon-γ 98. However, the behaviour of cells under the direct influence of bacteria remains less understood.

Reed *et al*. recently demonstrated that human embryonic stem cell-derived endothelial cells (hESC-ECs) are TLR4 deficient but respond to bacteria *via* the intracellular receptor nucleotide-binding oligomerization domain-containing protein 1 (NOD1). The authors suggested that hESC-ECs may be protected from unwanted TLR4-mediated vascular inflammation, thus offering a potential therapeutic advantage 99. On the other side, studies on DFPCs revealed the expression of TLR2 and TLR4 in both mRNA and protein level. Nevertheless, when these cells were treated with *P. gingivalis* LPS no effect on the expression of pro-inflammatory cytokines has been observed 47,78. Further, treatment with TLR4 agonist augmented the suppressive potential of DFPCs and increased the transforming growth factor-beta production 100. In accordance with these results canine ADSCs were shown to enhance immunomodulation after interacting with gastrointestinal microbes *in vitro* 101.

It is reported that LPS is able to induce the expression of the nuclear factor κB (NF-κB) -dependent gene IL-8 by DPSCs 102. He *et al*. suggested that LPS-mediated transcriptional and post-translational up-regulation of IL-8 in DPSCs is a process that also involves TLR4, myeloid differentiation primary response gene 88 (MyD88), NF-κB and mitogen-activated protein kinases 103. Further, Mei *et al*. demonstrated that MSCs improve the survival of sepsis by the down-regulation of inflammation-related genes (such e.g. IL-10 and IL-6) and a shift towards the up-regulation of genes involved in promoting phagocytosis and bacterial killing 104. The direct interaction of MSCs with the oral bacteria *F. nucleatum* and *P. gingivalis* led to a lower secretion of IL-8 compared to a differentiated tumour cell line 66. Raffaghello *et al*. support the immunomodulatory function of MSCs showing the inhibition of neutrophil apoptosis because of the secretion of IL-6 by MSCs 105. Nevertheless, these results should be interpreted carefully as it is speculated that the cytokine induction profile of stem cells is dependent on the cell type, bacterial species and methodology used (*e.g*. period of stimulation) 79,106.

## Current challenges and future perspectives

The rapid advancements in the field of dental research over the last few years could realize the promise of tissue regeneration through stem cells. Specifically, the demand for novel therapies against inflammatory diseases, like periodontitis, has created the need for a better understanding of the behaviour of the multipotent cells at sites of infection. Stem cells are supposed to support tissue homoeostasis by providing soluble factors, transdifferentiation or cell fusion 107. Hence, studies demonstrating stem cell responsiveness to bacteria raise questions on the possible contribution of multipotent cells to both tissue regeneration and outbreak of inflammation. Selected reports on the impact of bacteria on stem cells are listed in Table1.

**Table 1:** Selected reports on the impact of bacteria on stem cells

Biological impact	Cell populations	Bacterial species	Experimental model	Reference
Cell viability	DFPCs	*P. gingivalis*	Treatment with LPS	47
	PDLSCs	*P. gingivalis*	Treatment with LPS	53
	DFPCs, BMSCs	*P. gingivalis*	Treatment with LPS	78
	USSCs	Undefined	Treatment with LPS and flagellin	79
	DPSCs	*S. mutans*	Treatment with LPS	80
Differentiation	DFPCs	*P. gingivalis*	Treatment with LPS	47
	BMSCs	*E. coli*	Treatment with LPS	52
	PDLSCs	*P. gingivalis*	Treatment with LPS	53
	USSCs	Undefined	Treatment with LPS and flagellin	79
	DPPCs	*A.actinomyce-temcomitans*	Treatment with LPS	87
	DPSCs	*P. gingivalis*	Treatment with LPS	88
Immunomodulation	DFPCs	*P. gingivalis*	Treatment with LPS	47
	BMSCs	*P. gingivalis*, *F. nucleatum*, *A.actinomyce-temcomitans*	Co-culture model	69
	DFPCs, BMSCs	*P. gingivalis*, *F. nucleatum*	Co-culture model	70
	DFPCs, BMSCs	*P. gingivalis*	Treatment with LPS	78
	USSCs	Undefined	Treatment with LPS and flagellin	79
	ESC-ECs	Undefined	Treatment with LPS and *C12*-*iE*-*DAP*	99
	DFSCs, DPSCs	Undefined	Treatment with LPS	100
	AMSCs	*S. typhimurium*, *L. acidophilus*	Co-culture model	101
	DPSCs	*P. gingivalis*, *E. coli*, *P. endodontalis*	Treatment with LPS	102
	DPSCs	Undefined	Treatment with LPS	103
	MSCs	Undefined	Polymicrobial model of sepsis	104

Stem cells: AMSCs, adipose-derived mesenchymal stem cells; BMSCs, bone marrow stem cells; DFPCs, dental follicle progenitors cells; DPPCs, dental pulp progenitor cells; DPSCs, dental pulp stem cells; ESC-ECs, human embryonic stem cell-derived endothelial cells; MSCs, mesenchymal stem cells; PDLSCs, periodontal ligament stem cells; USSCs, unrestricted somatic stem cells. Bacteria: *A. actinomycetemcomitans*, *Aggregatibacter actinomycetemcomitans*; *E. coli*, *Escherichia coli*; *F. Prausnitzii*, *Faecalibacterium prausnitzii*; *L. acidophilus*, *Lactobacillus acidophilus*; *P. endodontalis*, *Porphyromonas endodontalis*; *P. gingivalis*, *Porphyromonas gingivalis*; *S. mutans*, *Streptococcus mutans*; *S. typhimurium*, *Salmonella typhimurium*.

The notion that bacteria may stimulate and drive the regenerative potential of stem cells should be further explored. Till now, data from *in vitro* studies utilizing single populations of cells challenged with bacterial components or mono-infections of planktonic bacteria may not adequately portray human periodontal diseases. Another significant parameter, which should be taken into consideration, is the oxygen concentration of *in vitro* models, as most of the pathogenic species implicated in the pathogenesis of periodontitis are obligate anaerobes. It is also remarkable that only few studies have used PDLSCs, which are the main population of multipotent cells residing within the periodontium. Thus, the development of new experimental settings to better resemble the *in vivo* periodontal milieu seems to be crucial.

A better understanding of the beneficial effects of bacteria on stem cells may allow future interventions based on cell priming with bacterial components prior to transplantation in sites of tissue destruction. Even the colonization of inflamed tissues with specific bacterial species that promote the mobilization of tissue-resident multipotent cell populations could be part of new therapeutic approaches. On the other side, the extent of stem cells’ involvement in immunomodulation remains to be clarified. Both immunosuppression and stimulation of host immune responses regulated by stem cells could be used as advanced tools against bacterially induced inflammation. In conclusion, the identification of intracellular signalling pathways regulating multipotency and immunomodulation of stem cells being exposed to bacteria may enable the development of successful therapeutic interventions in inflammatory diseases.

## Conflicts of interest

The authors confirm that there are no conflicts of interest.

## Author contributions

Kyriaki Chatzivasileiou and Katja Kriebel contributed substantially to the conception and design of the study and wrote the paper. Hermann Lang, Bernd Kreikemeyer and Gustav Steinhoff contributed to the conception and critical revision of the article and provided the final approval of the version to be published.
